# Consumer Rejection Threshold of Mung Bean Protein Hydrolysate: Unsweetened and Sweetened Brewed Teas as Test Models

**DOI:** 10.3390/foods15111875

**Published:** 2026-05-26

**Authors:** Kanokwan Promjeen, Niramon Utama-ang, Witoon Prinyawiwatkul

**Affiliations:** 1 School of Agro-Industry, Chiang Mai University, Chiang Mai 50100, Thailand; kanokwan_pr@cmu.ac.th (K.P.); niramon.u@cmu.ac.th (N.U.-a.); 2 School of Nutrition and Food Sciences, Agricultural Center, Louisiana State University, Baton Rouge, LA 70803, USA; 3 Sensory Evaluation and Consumer Testing Unit, Faculty of Agro-Industry, Chiang Mai University, Chiang Mai 50100, Thailand

**Keywords:** mung bean, protein hydrolysate, bitterness, sweetness, masking effects, consumer rejection threshold

## Abstract

Mung beans (*Vigna radiata* L.) can be considered an environmentally sustainable food due to their nutritional value, environmental benefits, and their potential in reducing reliance on animal-based proteins. Mung bean protein hydrolysate (MBPH) is a plant-based functional ingredient; however, its application in beverages is restricted by intense bitterness. This study was the first one to determine the consumer rejection threshold (CRT) of MBPH in three beverage matrices [water, unsweetened brewed tea (USBT), and sweetened brewed tea (SBT)] to evaluate how sweetness modulated bitterness perception and, in turn, affected consumer acceptance. Sensory evaluation was conducted with 308 consumers to evaluate acceptance of overall quality and bitter taste (yes/no), hedonic rating (overall liking and liking of taste and bitterness; a 9-point hedonic scale), and preference (a 2-alternative forced-choice, 2-AFC test) of three beverage matrices across MBPH concentrations of 0.0–1.2% (*w*/*v*). Acceptance decreased with increasing MBPH concentration across all matrices, with distinct differences in CRT values among samples. Based on overall acceptance, CRT values were 0.40% MBPH for water, 0.48% MBPH for USBT, and 0.80% MBPH for SBT. CRT values based on bitterness liking were lower (0.18–0.64%) compared to those (0.24–0.76%) based on overall taste and overall liking, indicating that bitterness perception was the primary driver of rejection. The 2-AFC results showed consistent preference for control samples; therefore, CRT could not be determined using this method under the experimental condition in this study. Overall, CRT values increased from 0.18–0.48% MBPH for USBT to 0.64–0.80% MBPH for SBT, demonstrating a quantitative shift associated with matrix composition and the presence of sweetness, providing a practical strategy for product developers to enhance the palatability of plant-based beverages containing MBPH.

## 1. Introduction

Plant-derived protein hydrolysates are increasingly used in food and beverages as functional ingredients due to their high digestibility, bioactive peptide content, and favorable techno-functional properties such as improved solubility, emulsification, and foaming [1]. However, their application is often limited by the bitter taste developed during enzymatic hydrolysis, which is primarily associated with the formation of low molecular-weight hydrophobic peptides [1,2,3]. This challenge is particularly relevant for mung bean protein hydrolysate (MBPH), which limits its acceptable incorporation level.

The bitter taste from peptides is a major sensory challenge across fish, dairy, and plant protein hydrolysates [1,2,4], and is influenced by the degree of hydrolysis, peptide size distribution, hydrophobicity, and specific amino acid sequences. Liu et al. [1] reported a bell-shaped relationship between bitterness and degree of hydrolysis, reflecting formation and degradation of hydrophobic peptides. Idowu and Benjakul [2] reported that bitterness was mainly driven by peptide hydrophobicity and chain length of fish protein hydrolysates. Many bitter peptides have low sensory taste thresholds, dominating flavor perception even at low concentrations [3], which highlight the need to define a practical limit for hydrolysate addition in food products [4].

Various technological strategies have been proposed to reduce or mask bitter taste, including selective removal of bitter peptides, enzymatic modification, and physical or colloidal methods that limit interactions with taste receptors [1,5,6,7]. Although effective under controlled conditions, these methods face challenges in beverage applications due to cost, regulatory issues, processing complexity, or impact on product identity [6]. Therefore, sensory-based approaches may serve as more practical alternatives to mask rather than completely remove bitter taste [1]. In a mixture, sweetness can suppress perceived bitterness, but the effect varies with the type of bitter compounds and sweeteners [8]. While sweetness improves palatability, bitterness suppression is rarely complete, and excessive sweetness may reduce acceptance or contradict the sugar-reduction goals [9]. Regardless of the approach being used, defining an acceptable incorporation level of protein hydrolysates in beverages based on consumer response remains a practical challenge.

Mung beans are an important leguminous crop due to their nutritional value and reported bioactive properties [10,11]. Similar to other plant protein hydrolysates, MBPH has been reported to exhibit antioxidant, anti-inflammatory, and immunomodulatory properties [12,13,14,15,16]. However, MBPH exhibits bitter and astringent sensory characteristics that may limit its use at physiologically relevant doses, particularly in beverage systems [12,13]. Tea-based beverages were selected as test model systems due to their widespread consumption [17]. Additionally, the inherent bitterness and astringency from polyphenols may interact with MBPH and sugar, which influence sensory perception. This interaction is particularly relevant in tea-based beverages, where bitterness can be modulated by added sweeteners, influencing overall sensory perception. To date, there have been no studies devoted to the determination of CRT of MBPH in food and beverage.

Therefore, this study aimed to determine the CRT of MBPH in different beverage matrices. It was hypothesized that the beverage matrix and the presence of sweetness would significantly influence CRT, with sweetened systems exhibiting higher tolerance to MBPH. CRT was determined using three complementary sensory approaches: overall acceptance (yes/no); hedonic ratings (a 9-point hedonic scale), and preference (a 2-AFC test). Three beverage matrices—water, USBT, and SBT—were used as test models to assess the extent to which sweetness influenced bitterness perception and consumer tolerance to MBPH.

## 2. Materials and Methods

### 2.1. Sample Preparation

MBPH was produced by enzymatic hydrolysis of mung bean (*Vigna radiata* L.) protein using Alcalase (Sigma Chemical Company, St. Louis, MO, USA), following a previously established laboratory protocol [13]. MBPH used in this study had a degree of hydrolysis of approximately 33% and consisted primarily of low-molecular-weight peptides, with major fractions at 1.1–10 kDa (45.57%) and <1 kDa (47.29%), as previously reported by Promjeen et al. [13].

Three beverage systems were used as test models: water, unsweetened brewed tea (USBT), and sweetened brewed tea (SBT). Brewed tea was prepared by heating 500 mL of purified drinking water (pH = 5.78; GreatValue^®^, Walmart, Bentonville, AR, USA) to 90–95 °C and steeping one commercial tea bag (Luzianne iced tea; New Orleans, LA, USA) for 5 min. After removal of the tea bag, 500 mL of cold purified drinking water was added to achieve a final volume of 1000 mL. USBT contained no added sugar, while SBT contained 10% (*w*/*v*) sucrose. The 10% sucrose concentration is frequently cited as a representative presweetened iced tea composition according to the USDA FoodData Central [18].

MBPH was incorporated into all beverage systems (water, USBT, and SBT) at concentrations of 0, 0.2, 0.4, 0.6, 0.8, 1.0, and 1.2% (*w*/*v*). The MBPH concentration range (0–1.2%, *w*/*v*) was selected to bracket a practical fortification level. Previous studies have demonstrated the biological activity of mung bean peptides in animal models at doses of 200–245 mg/kg/day. Based on the standard allometric scaling, these doses correspond to approximately 1.0–1.5 g/day in human-equivalent intake [19,20]. This intake level is approximately equivalent to 0.4–0.6% (*w*/*v*) in a 250 mL beverage serving. Therefore, the tested range was designed to include concentrations below, within, and above this practical range, enabling the identification of consumer rejection thresholds across different beverage matrices. All samples were stored at approximately 4 °C for 24 h prior to sensory evaluation.

### 2.2. Consumers, General Serving Conditions, and Sensory Methods

The sensory evaluation protocol involving human participants was reviewed and approved by the Louisiana State University (LSU) Agricultural Center Institutional Review Board (IRBAG-21-0063; Baton Rouge, LA, USA). A total of 308 participants were recruited from the LSU campus. Three independent consumer tests were conducted: water (n = 98), USBT (n = 105), and SBT (n = 105). All participants were between 18 and 60 years old, self-identified as regular tea consumers, and reported no allergies to legumes and tea products. To ensure participant safety and minimize the risk of allergic reactions, all participants were informed prior to the evaluation that the test samples contained MBPH. The exclusion criteria were people under 18 years old, pregnant women, and those who were allergic to the food ingredients used in this study. A consent form was e-signed by all participants to acknowledge the potential minimal risk associated with food allergens presented in this study.

Each test sample was portioned (20 mL) into a 1 oz plastic cup labeled with three-digit blinding codes and served at 8 ± 2 °C. Participants were not informed about the concentration levels or the order of sample presentation to avoid the psychological expectation error. Samples were presented in an ascending order of MBPH concentration (from 0% to 1.2% *w*/*v*) because the CRT method is a psychometric “dose–response” approach. Consumers were asked to indicate the point at which the product became unacceptable or rejected. To identify this point, the stimulus should increase gradually. If samples were randomized, consumers would taste the samples back and forth between low and high concentrations of MBPH, making it confusing to identify the first concentration that triggers rejection. A random order would increase noise and potential carryover effects associated with bitterness perception, hence reducing CRT precision. This approach is consistent with the standardized sensory threshold methodologies using ascending concentration series (ASTM E679-19 [21]; ASTM E1432-19 [22]), where samples are evaluated progressively from low to high concentration to identify the rejection point.

The concept of consumer rejection threshold (CRT) was applied to determine the concentration at which MBPH became unacceptable, based on a forced-choice method (2-AFC) initially proposed by Prescott et al. [23]. This approach enables the identification of practical formulation limits based on consumer preference rather than solely sensory detection [24]. However, in this current study, a series of sensory evaluations were sequentially conducted for each beverage matrix as follows: (a) acceptance: consumers indicated whether each sample was acceptable in terms of overall quality and bitterness (Yes or No); (b) hedonic ratings: consumers evaluated overall liking, overall taste liking, and bitterness liking using a 9-point hedonic scale (1 = dislike extremely; 5 = neither like nor dislike; 9 = like extremely); (c) preference: using the 2-AFC test, consumers were asked to compare each test sample containing MBPH with the reference sample without MBPH, and indicated which one they preferred [24,25].

Consumers were instructed to rinse their mouths with room-temperature purified drinking water between samples to cleanse their palate to reduce residual taste effects. Demographic information and sensory data were collected using an online questionnaire administered through Qualtrics (XM) survey software (Qualtrics, Provo, UT, USA). For each beverage matrix, the total evaluation time for each participant was approximately 10–15 min. All consumer tests were performed in the individual sensory booths equipped with LED cool white light under controlled conditions (ca. 23–25 °C and ca. 55–60% relative humidity) at the LSU Agricultural Center Sensory Services Laboratory (Baton Rouge, LA, USA). Consumers were not asked to use nose clips to mimic normal consumption. Each participant received the School of Nutrition and Food Sciences’ promotional item as an incentive after completion of the study.

### 2.3. Statistical Analysis and Determination of Consumer Rejection Threshold (CRT)

Given the objective of identifying population-level rejection thresholds, simpler statistical approaches were applied to facilitate interpretation and ensure consistency with established CRT methodologies [23,24,25].

For the acceptance data, the percentage of “yes” acceptance ratings of each sample was calculated. CRT was defined as the lowest MBPH concentration at which the % “yes” acceptance dropped below the critical (cut-off) value between 57 and 61%, depending on the sample size (n = 98–105) [24].

For the hedonic rating data, CRT was defined as the lowest MBPH concentration at which the mean hedonic rating was below the neutral category of 5 (neither like nor dislike); the resulting CRT does not account for any spread around the point-estimate [24]. Alternatively, CRT was calculated based on a one-sample *t*-test to estimate the point at which the mean hedonic rating (µ) was significantly (α = 0.05) below the 5.0 value. The one-sided hypothesis was as follows:

Ho: µ ≥ 5

Ha: µ < 5

A simple linear regression was used to model the calculated *t*-scores (*y*-axis) as a function of the MBPH level (*x*-axis). The calculated *t*-value was computed as follows:

$$t=(\bar{x}-5)/(\mathrm{SD}/\surd n),$$

where $\bar{x}$ and SD = a mean hedonic score and its associated standard deviation value, and n = the number of consumers. The critical *t*-value for a one-tailed test (α = 0.05; df = n − 1) was superimposed to identify CRTs. Specifically, the critical *t*-values were −1.66 for water (n = 98; df = 97) and −1.66 for both USBT and SBT (n = 105; df = 104). Mean hedonic ratings significantly lower than 5 (*p* < 0.05; one-tailed) were considered indicative of rejection, a more liberal CRT estimate.

For the preference data, the 2-AFC test was used to examine a shift in consumer preference relative to the control sample as the MBPH concentration increased. A binomial test was applied to evaluate whether the proportion of consumers preferring the control sample differed significantly (*p* < 0.05; one-tailed) from the chance expectation.

## 3. Results

### 3.1. CRTs Based on Acceptance Data

**Figure 1:**
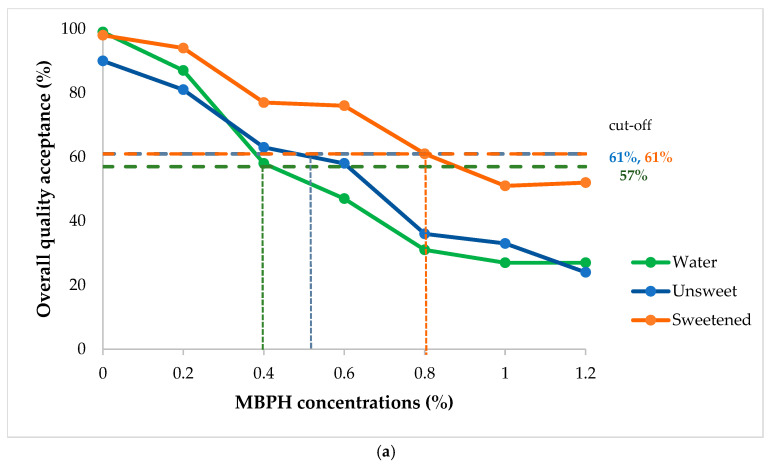

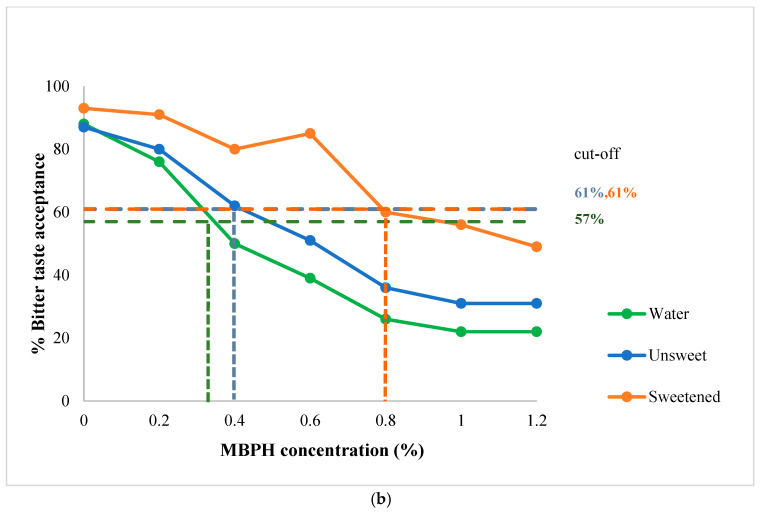
Consumer acceptance of (**a**) overall quality and (**b**) bitter taste of the MBPH-fortified beverage samples [Water, Unsweetened brew tea (USBT), and Sweetened brew tea (SBT)] as a function of MBPH concentration (%). Dashed lines represent the critical (cut-off) values: 57% and 61% for sample sizes of 98 and 105, respectively.

The observed decrease in consumer acceptance as the MBPH concentration increased was dose-dependent across all samples (Figure 1a,b). At low concentrations (≤0.2% *w*/*v* MBPH), all beverage samples maintained high overall acceptance (>80%) (Figure 1a). Beyond this concentration, acceptance trajectories diverged markedly across samples. Water exhibited the earliest decline, falling below the 57% cut-off threshold at approximately 0.40% MBPH, whereas USBT crossed its 61% threshold at approximately 0.48% MBPH. In contrast, SBT maintained acceptance above the cut-off threshold until approximately 0.80% MBPH, indicating substantially higher tolerance to MBPH. This represents an approximately two-fold increase in CRT for SBT compared to water and a ~1.7-fold increase compared to USBT. A comparable pattern was observed for bitter taste acceptance, where SBT maintained bitterness acceptance up to ~0.80% MBPH, while water and USBT crossed their thresholds at approximately 0.35–0.40% MBPH (Figure 1b).

### 3.2. CRTs Based on Hedonic Data

**Figure 2:**
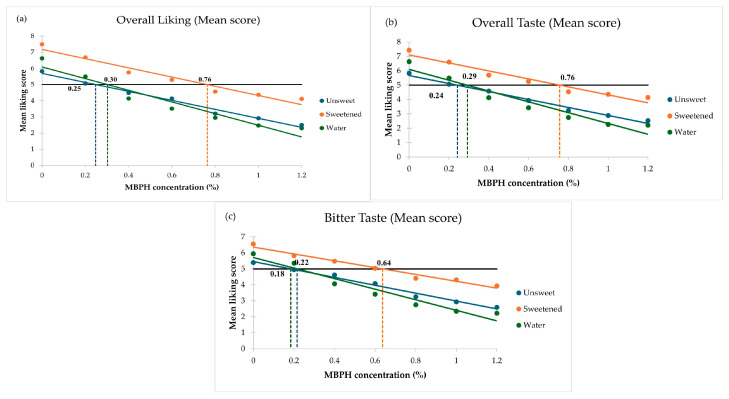
Mean liking scores for (**a**) Overall Liking, (**b**) Overall Taste, and (**c**) Bitter Taste of the MBPH-fortified beverage samples [Water, Unsweetened brew tea (USBT), and Sweetened brew tea (SBT)] as a function of MBPH concentration. The horizontal black line at a score of 5.0 (neither like nor dislike) represents a cut-off threshold. Vertical dashed lines indicate CRT, i.e., the specific concentration at which each matrix crosses this threshold.

The scores for overall liking (Figure 2a), taste liking (Figure 2b), and bitter taste liking (Figure 2c) decreased as the MBPH concentration increased.

Based on the overall liking scores, a steady dose-dependent decline was observed across all three beverage samples as the MBPH concentration increased (Figure 2a). Quantitatively, water and USBT were negatively more sensitive to MBPH fortification, crossing the neutral liking threshold (a score of 5) at 0.30% and 0.25% MBPH, respectively. In contrast, SBT showed a substantially higher tolerance, maintaining the mean overall liking scores (7.49) above the cut-off point (5.0) until reaching a concentration of 0.76%. This indicated that SBT increased tolerance to MBPH by approximately three-fold compared to that of USBT; this indicated that the combination of sweetness and tea flavor effectively maintained consumer liking even at a higher level of MBPH fortification (Figure 2a).

The results for overall taste mirrored the trends observed for overall liking, characterized by a steady decrease in mean scores as the concentration of MBPH increased (Figure 2b). The decline was most pronounced in the water and USBT samples, in which the scores fell below the cut-off threshold at 0.29% and 0.24% MBPH, respectively. SBT again demonstrated a higher tolerance to increasing MBPH concentration, in which the taste-liking scores remained above the cut-off threshold up to a concentration of 0.76% MBPH.

Analysis of bitter taste scores revealed that bitterness was a critical sensory barrier for MBPH incorporation. All samples crossed the cut-off thresholds earlier than observed for overall liking and overall taste modalities (Figure 2c). Water and USBT crossed the neutral bitterness threshold at lower concentrations (0.18% and 0.22% MBPH, respectively), whereas SBT reached this threshold at approximately 0.64% MBPH. This suggested that while sweetness delayed bitterness perception, it did not eliminate it. The perception of bitterness remained the limiting factor determining CRTs in all beverage samples. The observed bitterness can be partially attributed to the composition of MBPH, which consisted predominantly of low-molecular-weight peptides (<1 kDa and 1–10 kDa). Previous studies showed that such peptides, particularly those containing hydrophobic amino acids, contributed significantly to bitterness perception. Although hydrophobic amino acid composition was not quantified in this study, the relatively high degree of hydrolysis (~33%) suggested an increased presence of bitter-tasting peptides [13].

**Figure 3:**
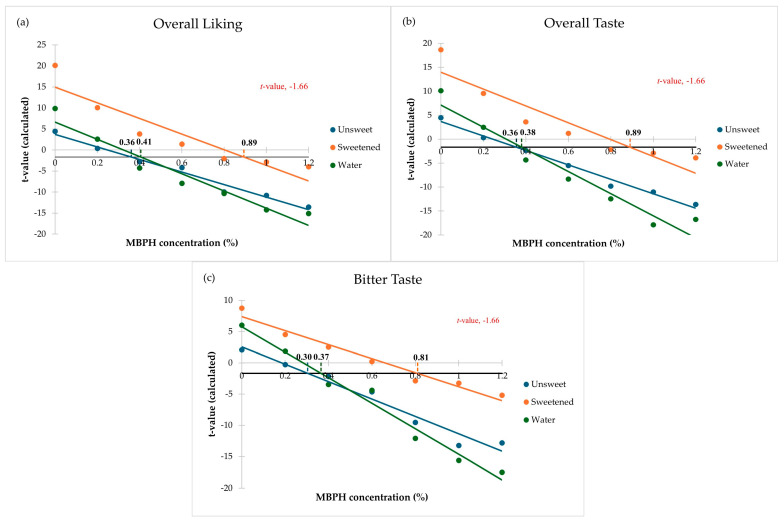
Calculated *t*-values for (**a**) Overall Liking, (**b**) Overall Taste Liking, and (**c**) Bitter Taste Liking across the three beverage samples [Water, Unsweetened brew tea (USBT), and Sweetened brew tea (SBT)] as a function of MBPH concentration. The horizontal line at *t*-value = 0 represents a point of neutral liking (a score of 5.0); points below this line indicate significant consumer disliking (*p* < 0.05). Values labeled on the line charts represent CRT, i.e., the specific MBPH concentration at which each sample crosses the critical *t*-value (one-tailed; α = 0.05; df = n − 1). Specifically, the critical *t*-values were −1.66 for water (n = 98; df = 97) and both USBT and SBT (n = 105; df = 104).

For a more liberal CRT estimate, the *t*-test analyses provided a robust statistical validation, identifying the MBPH concentration at which each beverage sample was rated significantly below the neutral liking score (*t*-value = 0) (Figure 3a–c). The obtained CRT values were slightly higher than those derived from a single cut-off point (a score of 5.0) as shown in Figure 2, as similarly observed by Ardoin et al. [24]. Across all sensory modalities, CRTs of SBT were about two times higher than those of USBT, indicating that the added sugar and tea flavor synergistically caused a positive shift in the CRT for MBPH.

### 3.3. 2-AFC Preference Shifts

**Figure 4:**
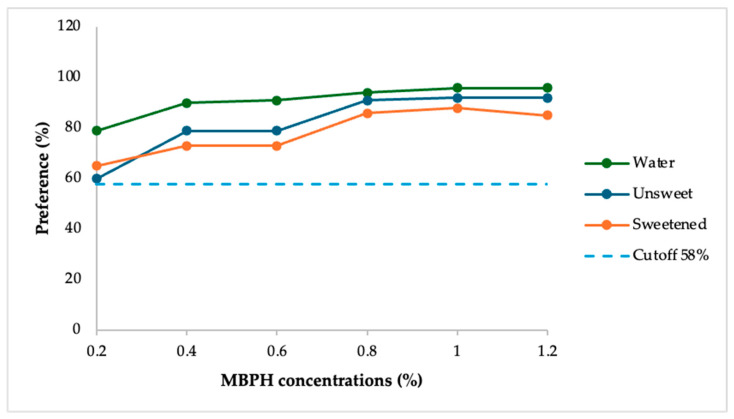
Percentage of consumers preferring the control (0% MBPH) over the MBPH-fortified samples [Water, Unsweetened brew tea (USBT), and Sweetened brew tea (SBT)] as a function of MBPH concentration. The dashed line at 58% represents the significant cut-off threshold for consumer preference (*p* < 0.05).

The 2-AFC preference test results indicated a clear dose-dependent shift in consumer choice as the concentration of MBPH increased, with most participants preferring the control (0% MBPH) over the MBPH-fortified samples (Figure 4). Even at the lowest concentration tested (0.2% MBPH), the preference for the control in the water sample was already high at approximately 80%, well above the 58% cut-off threshold. As the MBPH concentration rose to 1.2%, the preference for the control reached a peak of approximately 95% for water, 92% for USBT, and 85% for SBT.

Water exhibited the most immediate and significant (*p* < 0.05) preference for the control even at 0.2–0.4% MBPH, suggesting that in a neutral medium, consumers may detect bitter off-taste even at a low MBPH levels. At the higher concentrations (0.8–1.2%), preference for the control peaked at 90–95%, indicating a clear rejection of the MBPH-fortified sample due to bitter off-taste and possibly beany note [10,11]. A similar trend was observed for both USBT and SBT, with a lower % consumer preferring the control sample (0% MBPH) compared to the water sample (Figure 4). At 0.8–1.2% MBPH, the preference for the control over the MBPH-fortified samples was at 90–95%, 88–92%, and 82–85%, respectively, for water, USBT, and SBT. This indicated that sweetness in the SBT sample effectively masked bitterness of MBPH and delayed the onset of CRT.

One drawback of the 2-AFC preference test is that it is a forced-choice method, so consumers must select which sample of the two they prefer. As preference may not imply “acceptance” or “liking,” comparative assessments of preference do not always distinguish acceptable products from unacceptable ones [24]. As such, this method may not be suitable for determination of CRT under the test conditions in this current study. Although the preference-based outcomes were not used to define CRTs in the present study, the preference data provided a complementary perspective on consumer responses toward increasing MBPH concentration.

### 3.4. Comparison of CRTs from Acceptance and Hedonic Data

Regardless of the sensory methods and attributes investigated, the derived CRT (as in %MBPH) values followed a similar trend. The CRT values increased in the following order (high to low): SBT > USBT > water (Table 1). Based on the acceptance data (yes/no), the CRT value of SBT was about two times higher than that of USBT (0.80 vs. 0.40–0.48), while it was at least three times higher (0.64–0.76 vs. 0.18–0.25) when the liking scores based on a 9-point hedonic scale were used. This confirmed the bitterness masking effect of sugar in SBT.

## 4. Discussion

The present study utilized the CRT approach [24,26,27,28,29,30] to determine the maximum acceptable level of MBPH in tea-based beverage systems. Both acceptance and hedonic data consistently showed that increasing MBPH concentration led to a dose-dependent decrease in consumer acceptance and liking (Figure 1, Figure 2 and Figure 3), indicating that bitterness is the primary sensory driver of rejection. The observed decline in hedonic ratings with increasing MBPH concentration was consistent with previous studies on plant and dairy protein hydrolysates, where bitterness intensity strongly reduced consumer liking [15,25]. This effect is primarily attributed to the formation of low-molecular-weight, hydrophobic peptides during enzymatic hydrolysis, which are known to exhibit low taste thresholds and dominate sensory perception even at low concentrations [3,15]. The relatively high degree of hydrolysis (~33%) of MBPH [13] suggested a substantial presence of such bitter-tasting peptides.

Matrix effects played a critical role in modulating consumer response to MBPH with the CRT values following the order (high to low) SBT > USBT > water (Table 1), indicating that more complex matrices increased tolerance to bitterness. In water, which lacks competing sensory stimuli, it provides little room for flavor interactions or masking, so the bitter note of MBPH was fully expressed, leading to the lowest CRT. This observation was consistent with previous studies reporting that hydrolysates in simple matrices such as water tend to overestimate sensory impact compared with a more complex food system [25]. In contrast, tea-based matrices provided intrinsic flavor and aroma compounds, including polyphenols that contribute bitterness and astringency, which may interact with MBPH and alter overall perception through sensory integration. The higher tolerance for MBPH in SBT compared with water and USBT could be interpreted through sweetness–bitterness interactions and matrix effects, as reflected in the observed positive shift in the CRT values.

Recent work on plant-protein beverages and plant-based foods confirmed that sweetness and congruent flavor cues often suppress bitterness and shift attention toward more pleasant taste qualities, especially in complex matrices such as teas or dairy analogues [25,31,32]. Ardoin et al. [26] showed that complex matrices can attenuate or reshape bitterness from phenolic-rich ingredients and added protein hydrolysates. The matrix composition and existing taste–aroma profiles could modulate the salience of bitter tastes and could dilute or partially mask bitterness from functional ingredients [25,26,33].

Between the two tea matrices, SBT consistently showed higher overall liking scores and a higher CRT (% MBPH) than USBT. Within the MBPH concentration range tested, many consumers continued to rate SBT samples as acceptable with overall liking scores > 5.0 even when bitterness increased, provided that the sweetness perception supposedly remained sufficiently high. This finding supports the hypothesis that sweetness suppresses bitterness in MBPH-fortified tea beverages, and is consistent with the work on taste–taste interactions, where sucrose and other sweeteners reduced the perceived bitterness of caffeine, quinine-like compounds, and other bitterants in beverages and model systems [33,34]. From a formulation standpoint, without additional masking strategies such as sweetening, aroma design, Maillard-type flavor generation, or encapsulation, MBPH levels may need to be kept below CRTs while attempting to achieve targeted bioactivity [1]. Although added sucrose or other sweeteners delay CRTs, this strategy must be balanced against sugar-reduction efforts. Recent work highlighted that non-nutritive sweeteners, aroma-induced sweetness enhancement, or sugar–acid combinations can be used to reduce added sugar while maintaining masking efficiency [6,31].

The mechanistic T1R/T2R interaction was not investigated in this study. However, the bitterness suppression in SBT could be speculated based on previous work. During gustatory processing, bitter peptides activate bitter taste receptors (T2Rs), whereas sucrose activates sweet taste receptors (T1R2/T1R3) [35]. When these stimuli are perceived simultaneously, the gustatory system integrates competing signals, resulting in a reduction in the perceived combined intensity [34,36]. This mechanism increased consumer tolerance toward bitterness rather than eliminating bitterness itself, explaining the higher CRT observed in SBT compared with USBT matrices (Table 1).

Advances in debittering technologies [1,7,37,38] for protein hydrolysates can broaden the food applications of MBPH. In tea beverages, specifically, whey protein isolate-β-cyclodextrin nanocomplexes masked the bitter/astringent taste of EGCG and enhanced its stability, illustrating the feasibility of colloidal designs for MBPH-fortified teas [31]. Encapsulation of legume hydrolysates into biopolymer gels or high-internal-phase emulsions likewise reduced bitterness and astringency while maintaining enzyme-inhibitory bioactivity, demonstrating that structural design of the carrier phase can substantially shift sensory acceptance [32]. Although a debittering treatment was not applied to MBPH in the present study, it would have yielded higher CRTs in water or USBT samples destined for low- or no-sugar functional beverages. Future studies should be conducted to prove this postulation.

From a formulation perspective, the approximately two- to three-fold increase in CRT in SBT compared with water and USBT highlighted the importance of matrix design in improving the palatability of protein-fortified beverages. These findings suggested that incorporating MBPH into systems with inherent sweetness or complementary flavor profiles can significantly expand the acceptable MBPH concentration range. Finally, despite the dose-dependent decline in liking scores across three sample matrices (Figure 2 and Figure 3), the dispersion of scores (SD ≈ 1.6–2.3 based on data from Figure 2 and Figure 3) tentatively indicated substantial inter-individual variability in bitterness tolerance and acceptance. This variability highlighted a critical challenge in fortifying beverages with plant-based protein hydrolysates to be developed for a specific consumer segment and should be further investigated (see Supplementary Materials for exploratory segmentation analysis).

Overall, these findings demonstrated that CRT was strongly influenced by both matrix composition and sensory interactions. By leveraging sweetness and complex flavor systems, product developers can increase consumer tolerance to bitter functional ingredients such as MBPH, thereby improving the feasibility of developing nutritionally enhanced beverages.

## 5. Conclusions, Limitations, and Future Studies

This study demonstrated that the CRT of MBPH was strongly influenced by beverage matrix, with sweetness significantly increasing tolerance to bitterness. Among the tested systems, water showed the lowest tolerance, while SBT exhibited the highest CRT values, highlighting the role of matrix composition in modulating sensory perception. Importantly, the integration of multiple sensory approaches (acceptance, hedonic, and preference tests) provided a comprehensive understanding of consumer responses. While preference data from the 2-AFC method confirmed a strong consumer preference pattern toward the control, acceptance and hedonic measures were more appropriate for defining CRT. Together, these approaches demonstrated that bitterness remained the primary limiting factor for MBPH incorporation across all sample matrices.

From an application standpoint, the results highlight the importance of matrix design in expanding the acceptable usage level of MBPH in beverages. Incorporating MBPH into systems with sweetness or complex flavor profiles can increase tolerance to MBPH by approximately two to three times, offering a practical strategy for improving sensory acceptance. However, such approaches must be balanced with health and nutritional considerations, particularly in relation to added sugar.

A limitation of this study was the lack of detailed peptide characterization, which restricted direct linkage between chemical composition, sensory perception, and CRT values. Generalization regarding the effects of sweetness on bitterness suppression of MBPH should be cautiously made, and other beverage models should also be explored to confirm the findings from this study. Future research should systematically integrate compositional analysis with sensory descriptive analysis to characterize bitterness, astringency, and related flavor attributes, and to establish relationships between chemical constituents (e.g., peptides, phenolics) and sensory perception. This effort, along with the exploration of alternative bitterness-masking strategies, would further support the development of low-sugar functional beverages containing MBPH.

Overall, this study provided practical methodological guidance for defining acceptable incorporation levels of MBPH and demonstrated the usefulness of the CRT approach as a tool for product development in plant-based functional beverages.

## Figures and Tables

**Table 1:** Comparison of consumer rejection threshold (as in % MBPH) derived from acceptance and hedonic data for three beverage samples.

Samples	Acceptance of Overall Quality	Acceptance of Bitter Taste	Liking Scores		
			Overall Liking	Overall Taste	Bitter Taste
Water	0.40	0.35	0.30	0.29	0.22
Unsweetened brewed tea	0.48	0.40	0.25	0.24	0.18
Sweetened brewed tea	0.80	0.80	0.76	0.76	0.64

## Data Availability

The original contributions presented in the study are included in the article/Supplementary Material, further inquiries can be directed to the corresponding author.

## Supplementary Materials

The following supporting information can be downloaded at: https://www.mdpi.com/article/10.3390/foods15111875/s1, S1. Preliminary Consumer Segmentation Method, S2. Results: Preliminary Consumer Segmentation Based on Liking Responses, Figure S1. Cluster profile plots of standardized liking scores across MBPH concentrations in (a) water, (b) USBT, and (c) SBT. Each line represents the centroid values of each cluster, illustrating distinct consumer response patterns and matrix-dependent variations in MBPH tolerance.
