# Supplementary material for: Consumer Rejection Threshold of Mung Bean Protein Hydrolysate: Unsweetened and Sweetened Brewed Teas as Test Models
Source: Foods. 2026 May 26;15(11):1875. doi: 10.3390/foods15111875 (PMC13256719; doi:10.3390/foods15111875)
Supplement: Supplementary file 1 [file foods-15-01875-s001.zip › foods-4273513-supplementary.pdf]

Supplementary

S1. Preliminary Consumer Segmentation Method

Consumer segmentation was conducted using the K-means clustering method based on overall liking scores collected across MBPH concentrations for each sample matrix (water, USBT, and SBT). Prior to clustering, liking scores were standardized to minimize scale differences across individuals. Clustering analysis was performed separately for each matrix to capture matrix-dependent variations in consumer response. The optimal number of clusters was determined based on interpretability and consistency of clustering patterns, and a three-cluster solution (low, moderate, and high acceptance groups) was selected for all matrices.

The K-means algorithm was implemented using the Euclidean distance to assign observations to clusters. The algorithm was initialized with random seeds and iterated until convergence was achieved. Cluster centroids were used to generate profile plots, illustrating changes in liking scores across MBPH concentrations for each consumer segment. The distribution of participants across clusters was also examined to assess differences in consumer tolerance toward MBPH among matrices.

This exploratory analysis was intended to provide additional insight into inter-individual variability and to complement the population-level interpretation derived from CRT analysis. Cluster solutions were evaluated based on interpretability and stability of cluster patterns. All analyses were performed using jamovi version 2.6.45.0.

S2. Results: Preliminary Consumer Segmentation Based on Liking Responses

The K-means clustering analysis was conducted separately for each beverage matrix (water, USBT, and SBT) based on overall liking scores across MBPH concentrations. A three-cluster solution was selected in all cases, revealing distinct consumer segments with varying tolerance levels toward MBPH. For the water samples, three clusters were identified, including a low-acceptance group (n = 46), a moderate-acceptance group (n = 37) and a high-acceptance group (n = 15). The low-acceptance group exhibited consistently negative liking scores across all concentrations, indicating strong rejection of MBPH, whereas the high-acceptance group showed positive responses even at higher concentrations. In USBT, a shift in consumer response patterns was observed compared with water; the presence of tea appeared to partially mitigate the negative perception of MBPH. In SBT, consumer acceptance further increased, with the largest group classified as high acceptors (n = 38), followed by moderate- (n = 42) and low-acceptance groups (n = 16). This distribution indicated that sweetness significantly enhanced consumer tolerance toward MBPH. Overall, clustering analysis highlighted heterogeneity in consumer responses and revealed a shift in consumer segments across matrices, from predominantly low acceptance in water to higher acceptance in SBT. These findings further supported the hypothesis that sweetness and the beverage matrix significantly influenced consumer rejection behavior and highlighted the importance of considering consumer heterogeneity in sensory evaluation.

Cluster profile plots revealed distinct consumer response patterns across beverage matrices. In water, three clusters were clearly differentiated, with Cluster 1 showing increasing liking with MBPH concentration, Cluster 2 exhibiting consistent rejection, and Cluster 3 displaying decreasing liking at higher concentrations. In USBT, a similar

 https://doi.org/10.3390/foods15111875

segmentation pattern was observed; however, the negative impact of MBPH on liking was more pronounced, particularly for Cluster 2 and Cluster 3, suggesting enhanced bitterness perception in the tea matrix. In SBT, although clustering patterns remained comparable to USBT, the decline in liking was less pronounced, indicating that the presence of sugar partially mitigated the negative sensory impact of MBPH. These clustering results complemented CRT findings by demonstrating that rejection thresholds were not uniform across consumers in this current study. While CRT provided a population-level estimate of acceptability, clustering analysis revealed that some consumer segments may tolerate MBPH at levels beyond the identified CRT, whereas others may reject it at substantially lower concentrations. This highlighted the importance of considering both threshold-based and segmentation-based approaches for product optimization. These patterns indicated that consumer responses to MBPH were not uniform across individuals. In particular, certain clusters maintained acceptable liking scores at higher MBPH concentrations, suggesting that CRT values derived at the population level may underestimate tolerance in specific consumer segments. This observation further supported the importance of considering inter-individual variability in product formulation.

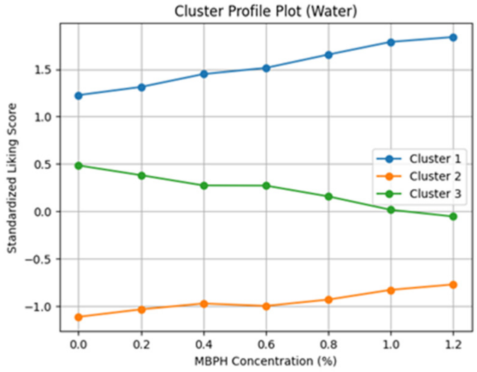

(a)

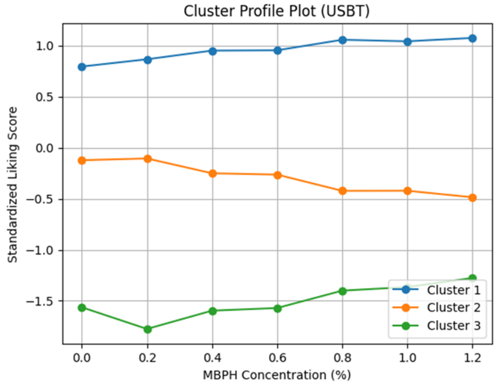

(b)

https://doi.org/10.3390/foods15111875

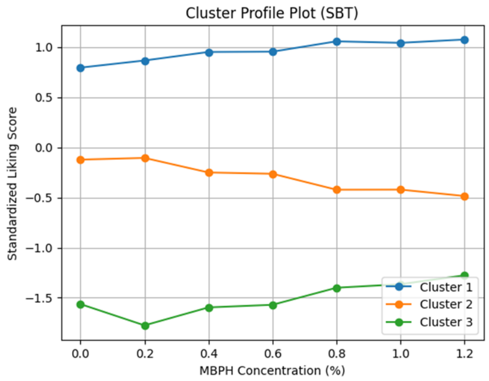

(c)

Figure S1. Cluster profile plots of standardized liking scores across MBPH concentrations in (a) water, (b) USBT, and (c) SBT. Each line represents the centroid values of each cluster, illustrating distinct consumer response patterns and matrix-dependent variations in MBPH tolerance.

https://doi.org/10.3390/foods15111875
